# Supplementary figures and images for: Sinking of microbial-associated microplastics in natural waters
Source: PLoS One. 2020 Feb 3;15(2):e0228209. doi: 10.1371/journal.pone.0228209 (PMC6996825; doi:10.1371/journal.pone.0228209)

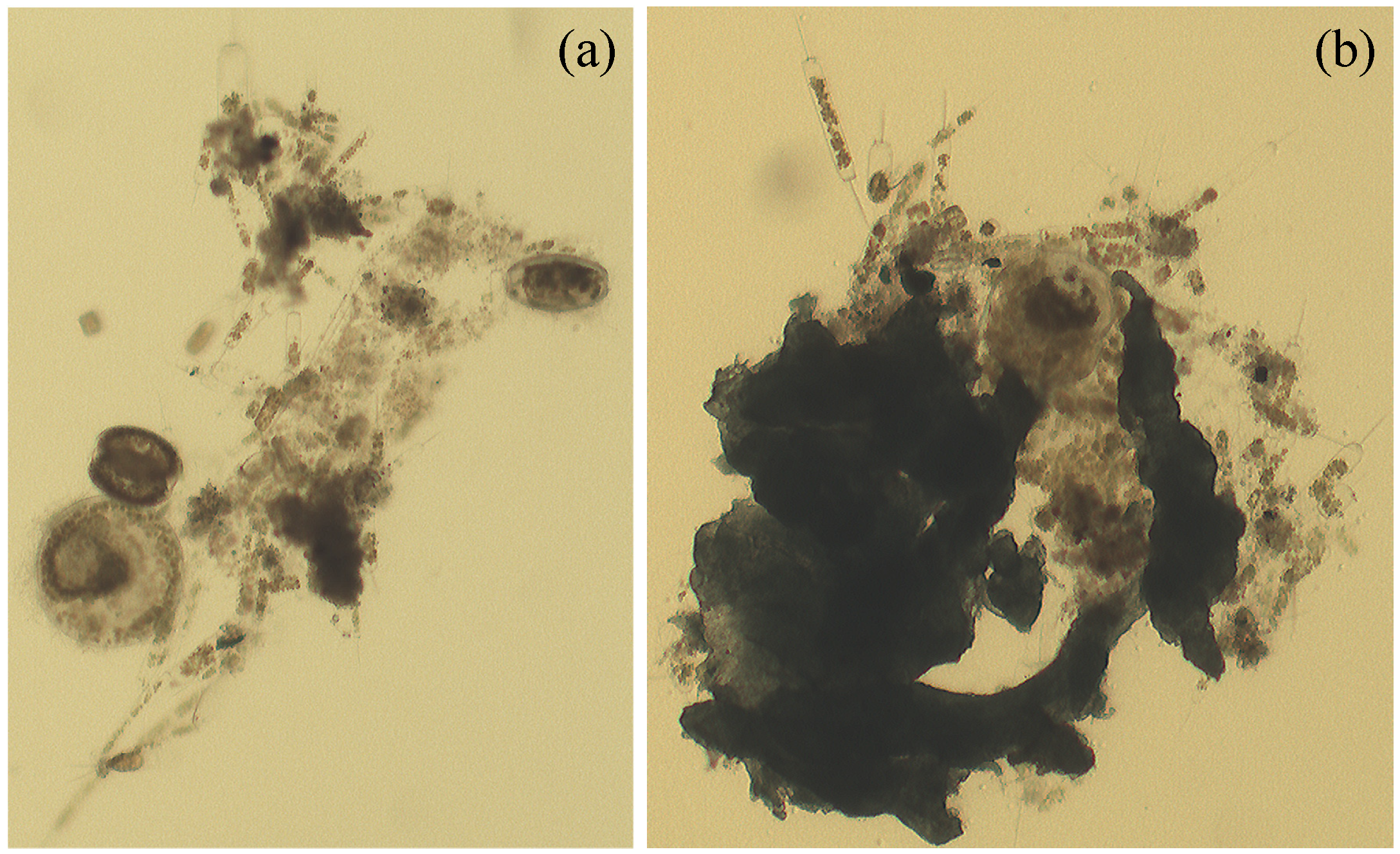

Supplement: S1 Fig — (a) Microorganisms sampled at Hawkesbury River, NSW, Australia after 2 weeks of pre-incubation, and (b) an aggregate of blue polyurethane microplastics and microorganisms from (a). Images were acquired using a Motic® series BA310 microscope at 10X magnification. (TIF) [file pone.0228209.s001.tif]
